# Expression of the preadipocyte marker ZFP423 is dysregulated between well-differentiated and dedifferentiated liposarcoma

**DOI:** 10.1186/s12885-022-09379-6

**Published:** 2022-03-21

**Authors:** Thanh N. Dang, Rafael P. Tiongco, Loren M. Brown, Jessica L. Taylor, John M. Lyons, Frank H. Lau, Z. Elizabeth Floyd

**Affiliations:** 1 Pennington Biomedical Research Center, Baton Rouge, Louisiana 70808 USA; 2 Tulane University School of Medicine, New Orleans, Louisiana 70118 USA; 3 Department of Surgery, Louisiana State University Health Science Center, New Orleans, Louisiana 70112 USA; 4 Our Lady of the Lake Medical Center, Baton Rouge, Louisiana 70808 USA

**Keywords:** Liposarcoma, SIAH2, ZFP521, ZFP423, Adipogenesis, PPARgamma

## Abstract

**Background:**

Well-differentiated and dedifferentiated liposarcomas are rare soft tissue tumors originating in adipose tissue that share genetic abnormalities but have significantly different metastatic potential. Dedifferentiated liposarcoma (DDLPS) is highly aggressive and has an overall 5-year survival rate of 30% as compared to 90% for well-differentiated liposarcoma (WDLPS). This discrepancy may be connected to their potential to form adipocytes, where WDLPS is adipogenic but DDLPS is adipogenic-impaired. Normal adipogenesis requires Zinc Finger Protein 423 (ZFP423), a transcriptional coregulator of Perixosome Proliferator Activated Receptor gamma (*PPARG2*) mRNA expression that defines committed preadipocytes. Expression of ZFP423 in preadipocytes is promoted by Seven-In-Absentia Homolog 2 (SIAH2)-mediated degradation of Zinc Finger Protein 521 (ZFP521). This study investigated the potential role of ZFP423, SIAH2 and ZFP521 in the adipogenic potential of WDLPS and DDLPS.

**Methods:**

Human WDLPS and DDLPS fresh and paraffin-embedded tissues were used to assess the gene and protein expression of proadipogenic regulators. In parallel, normal adipose tissue stromal cells along with WDLPS and DDLPS cell lines were cultured, genetically modified, and induced to undergo adipogenesis in vitro*.*

**Results:**

Impaired adipogenic potential in DDLPS was associated with reduced ZFP423 protein levels in parallel with reduced PPARG2 expression, potentially involving regulation of ZFP521. SIAH2 protein levels did not define a clear distinction related to adipogenesis in these liposarcomas. However, in primary tumor specimens, *SIAH2* mRNA was consistently upregulated in DDLPS compared to WDLPS when assayed by fluorescence in situ hybridization or real-time PCR.

**Conclusions:**

These data provide novel insights into ZFP423 expression in adipogenic regulation between WDLPS and DDLPS adipocytic tumor development. The data also introduces *SIAH2* mRNA levels as a possible molecular marker to distinguish between WDLPS and DDLPS.

**Supplementary Information:**

The online version contains supplementary material available at 10.1186/s12885-022-09379-6.

## Background

Liposarcomas (LPS) are a group of adipocytic tumors of mesenchymal cell origin. They are the most common type of soft tissue sarcoma and are classified into myxoid/round cells, pleomorphic, well-differentiated (WDLPS), or dedifferentiated (DDLPS) neoplasms based on their molecular and histological characteristics. Of the four LPS subtypes, WDLPS is the most common, constituting approximately 45% of all liposarcomas [1]. Up to 10% of WDLPS can convert to DDLPS, a more aggressive form of liposarcoma with higher metastatic potential. But DDLPS development is not exclusively dependent on WDLPS and can arise de novo [1]. The occurrence of DDLPS substantially decreases the 5-year survival expectancy to only 30% compared to 90% with WDLPS [1]. Treatments for WDLPS and DDLPS are currently limited and most chemotherapeutic treatments have low efficacy, leaving surgical excision and radiation treatments as the preferred options [1, 2].

Unlike the formation of adipocytes in WDLPS, terminal differentiation to adipocytes is impaired in DDLPS. The difference in adipogenic potential between WDLPS and DDLPS is a key clinical diagnostic feature consistent with the favorable prognosis of WDLPS compared to DDLPS [1, 3, 4]. The improved clinical outcome associated with forming adipocytes in the WDLPS has prompted studies focused on stimulating adipocyte formation in the DDLPS as a therapeutic option [5–8].

Tontonoz et al [5] exploited the role of the adipocyte-specific isoform of the nuclear receptor peroxisome proliferator-activated receptor gamma (PPARG2) in terminal differentiation of adipocytes to investigate adipocyte development in liposarcomas. PPARG2 activation is required for converting adipocyte precursor cells to adipocytes [9–13] and promising results were obtained in vitro using well-known PPARG agonists. Although PPARG2 activation was associated with reduced cell proliferation and increased lipid accumulation, stimulating PPARG2 activity has not consistently improved clinical outcome in phase II clinical trials [6, 7]. Nonetheless, recent preclinical evidence supports promoting adipogenesis in DDLPS as a viable approach to limit the tumorigenicity of DDLPS cells [8]. Those results suggest steps indirectly related to ligand-dependent activation of PPARG2 in converting adipocyte precursors to mature adipocytes may represent a therapeutic target. However, the molecular events underpinning the differentiation defect in DDLPS or WDLPS are poorly understood.

*PPARG2* mRNA expression in normal adipose tissue is determined by by Zinc-finger protein 423 (ZFP423), a protein that identifies committed preadipocytes [14, 15] and functions as a transcriptional co-activator of the adipocyte-specific *PPARG2*, but not the non-specific *PPARG1* isoform [14]. In turn, ZFP423 is upregulated by the pro-adipogenic factor, Early B-cell Factor 1 (EBF1), and directly repressed by the anti-adipogenic factor, Zinc-finger protein 521 (ZFP521) [16–18]. Several studies demonstrated precursor cell commitment to adipogenesis is directed by the relative expression level of EBF1 and ZFP521 proteins, where reduced ZFP521 levels are associated with adipocyte differentiation [17–19].

The ubiquitin ligase Seven-in absentia homolog 2 (SIAH2) is a RING-type ubiquitin ligase well-described as an oncoprotein that promotes metastasis of breast, melanoma and prostate cancer [20–22], likely via regulation of hypoxic responses that characterize tumorigenicity [23]. We found that *SIAH2* mRNA is expressed in PDGFRα^+^ and SCA-1^+^ adipocyte precursor cells, positioning SIAH2 as a potential regulatory factor in converting adipocyte precursor cells to mature adipocytes [24]. Our studies also showed that SIAH2 promotes expression of ZFP423 and adipogenesis [24–27] by targeting ZFP521 for ubiquitin-proteasome degradation in preadipocytes [24, 27] . SIAH2’s roles in adipogenesis and tumor development suggest that factors in adipocyte precursor cells controlling *PPARG2* mRNA expression are potentially dysregulated in the development of DDLPS.

In this study, we examined ZFP423, SIAH2 and ZFP521 expression in WDLPS and DDLPS tissues and during induction of adipogenesis in WDLPS and DDLPS cell lines along with a panel of other markers of adipogenesis. The current data indicates adipogenesis in DDLPS is dysregulated upstream of PPARG2, likely at regulation of ZFP423 protein expression. This is consistent with divergence of WDLPS and DDLPS adipogenic potential at preadipocyte commitment. Although SIAH2 protein expression did not show a consistent pattern between WDLPS and DDLPS in the tissues and cell lines, *SIAH2* mRNA is highly expressed in paraffin-embedded DDLPS tissue stromal cells, colocalizes with DDLPS tumor-associated macrophages and is expressed at higher levels in DDLPS compared to WDLPS tissues. Overall, our results shed new light on differences between WDLPS and DDLPS in expression of factors that control the conversion of adipocyte precursor cells to mature adipocytes.

## Methods

### Surgical tissues

Tissues were obtained from four sources: the Sarcoma Alliance for Research through Collaboration (SARC) provided paraffin-embedded liposarcoma tissues, the Biospecimen Core Laboratory of the Louisiana Cancer Research Center provided paraffin-embedded and frozen tissues. Fresh WDLPS and DDLPS tissues were obtained from Our Lady of the Lake Regional Medical Center (OLOLMC, Baton Rouge, LA) and normal retroperitoneal white adipose tissue (rpWAT) was provided by the LSU Health Science Center (New Orleans, LA). All liposarcoma tissues were classified by a pathologist. A total of 19 samples was examined, consisting of six healthy retroperitoneal adipose tissues (three frozen, three paraffin-embedded), six well-differentiated liposarcoma tissues (two fresh, four paraffin-embedded), and seven dedifferentiated liposarcoma tissues (two fresh, five paraffin-embedded). Fresh tissue collections were approved by the LSUHSC-New Orleans (LSUHSC-NO IRB, IRB#10296) and the Franciscan Missionaries for Our Lady University-OLOLMC (IRB#2020–026).

### Cell culture

Normal human retroperitoneal adipose stromal cells (HuASC) were provided by Dr. Frank Lau. Lipo863, Lipo224, Lipo815 DDLPS cell lines were obtained from MD Anderson Cancer Center (Cytogenetics and Cell Authentication Core). The lifetime passage number for the DDLPS cell lines frozen stocks was 41–65. The cells were used in experiments at passage 3–6 after plating the frozen stock. The well-differentiated liposarcoma 94 T778 cell line was purchased from ATCC (CRL-3044) and used at passage number 3–6 from the frozen stock. The primary HuASCs were used at passage number 2–3. Liposarcoma cell lines were cultured in RPMI-1640 medium, 10% FBS, and 100 units penicillin/100 μg streptomycin. HuASC cells were cultured in DMEM/F12 medium, 10% FBS, and 100 units penicillin/100 μg streptomycin. When cell growth reached 90% confluence, adipogenesis was induced using a differentiation cocktail (3% characterized FBS, 100 units penicillin/100 μg streptomycin, 1 μM dexamethasone, 500 μM IBMX, 33 μM biotin, 5 μM rosiglitazone, 100 nM insulin, and 17 μM panthothenate in RPMI-1640 medium for liposarcoma cell lines or DMEM/F12 medium for HuASCs). After 72 h, the media was exchanged to maintenance media (differentiation media minus IBMX and rosiglitazone). At the indicated time-point, the cells were rinsed in ice cold phosphate-buffered saline, pH 7.4 (PBS) and collected in RIPA buffer (50 mM Tris-Cl pH 8.0 with 150 mM NaCl, 1% NP-40, 0.5% sodium deoxycholate, 0.1% sodium dodecyl sulfate (SDS), 1 μM phenylmethylsulfonyl fluoride, 1 μM pepstatin, 50 trypsin inhibitory milliunits of aprotinin, 10 μM leupeptin, and freshly prepared 10 mM N-ethyl maleimide) for total protein extraction or TriReagent for RNA extraction.

### *ZFP423* siRNA knockdown and retroviral-mediated overexpression

*ZFP423* expression was depleted in the HuASC and DDLPS Lipo863 cells using human *ZFP423* siRNA alongside a siRNA negative control according to the manufacturer’s protocol (Cat#: 43924 and 4,390,843, respectively, ThermoFisher). HuASC and DDLPS Lipo863 cells were transfected with the siRNA construct using Lipofectamine RNAiMAX (ThermoFisher, Cat#: 1377–030). After 72 h, transfected cells were induced for adipocyte differentiation as outlined in Cell culture. At day four post-induction, the cells were rinsed in ice cold PBS and collected in RIPA buffer (see Cell culture for composition) for total protein extraction or TriReagent for RNA extraction.

*ZFP423* overexpression was performed in the WDLPS and DDLPS Lipo224 cell lines by retroviral infection using pMSCVFLAG-ZFP423 plasmid (Addgene, Cat. #: 24764) or the empty pMSCV vector (Addgene, Cat. #: 47539) as a negative control. The retroviral particles were produced in Phoenix-AMPHO cells (ATCC, Cat #: CRL-3213) transfected with either pMSCVFLAG-ZFP423 vector or the empty pMSCV vector using a CaPO_4_ precipitation method and processed as described [28].

The targeted cells were plated and infected with retroviral particles in the presence of 5 μg/mL polybrene. The media were changed 24 h post-infection and puromycin (2.5 μg/mL) selection was initiated at 80% confluency and maintained for 2 weeks. Puromycin was removed prior to induction of adipocyte differentiation as outlined in Cell culture. At day ten post induction, the cells were rinsed in ice cold PBS and collected in RIPA buffer for total protein extraction or TriReagent for RNA extraction.

### Gene expression analysis

**Fig. 1:**
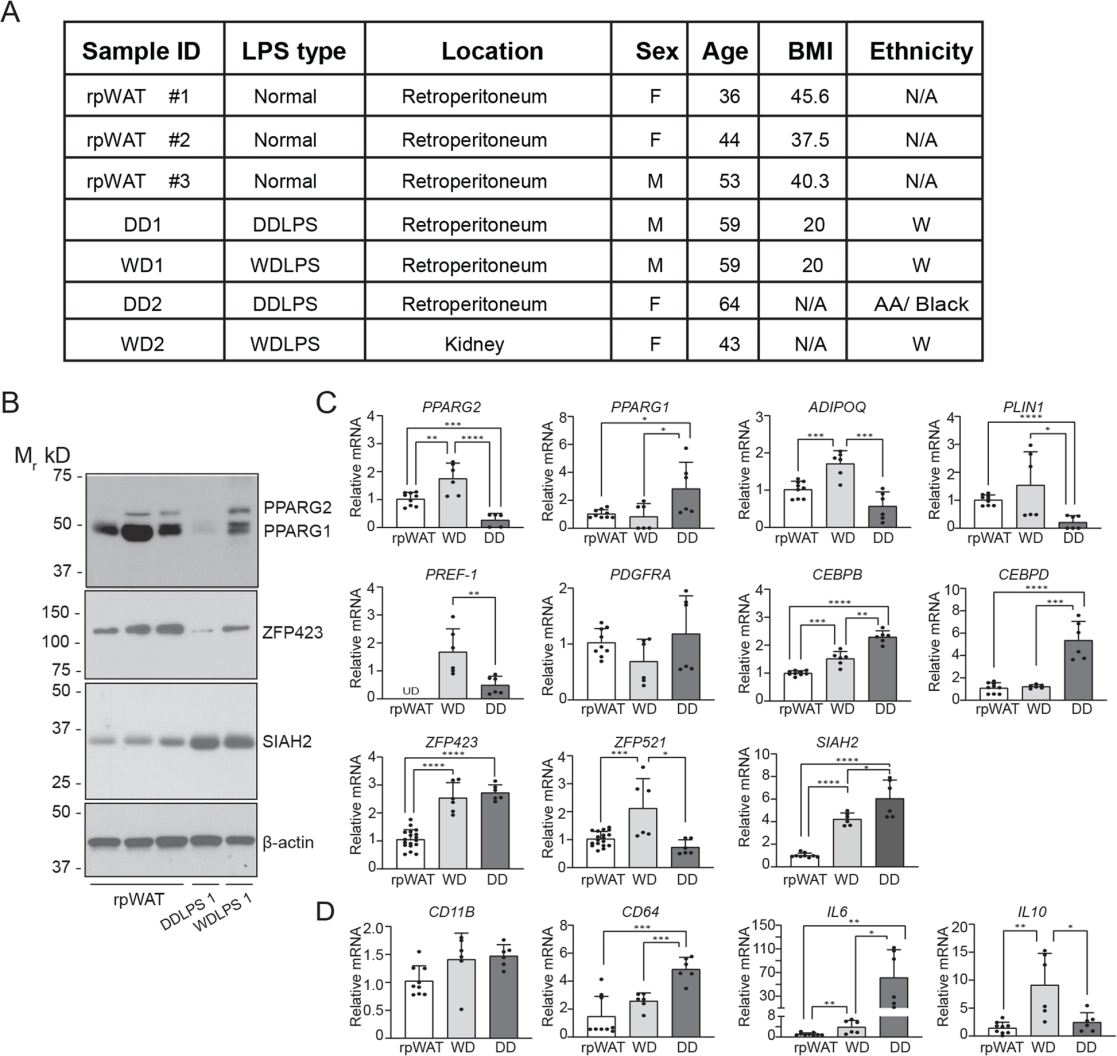
Adipogenesis in LPS tissues is dysregulated at preadipocyte commitment. **A** Demographic information available for tissues included in the analysis. **B** Western blots analysis of SIAH2, ZFP423, PPARG, and β-actin. Biological replicates of western blot analyses are available in Supplemental Fig. S1, along with full-length original western blots and densitometry ratio quantifications. **C** Gene expression of markers of mature adipocytes (*PPARG2*, *ADIPOQ*, *PLIN1*), preadipocyte markers (*PREF-1*, *PDGFRA*, *ZFP423*) or regulatory factors involved in adipogenesis (*CEBPB*, *CEBPD*, *ZFP521*, *SIAH2*). **D** Gene expression of macrophage *(CD11B*, *CD64*) and cytokine (*IL6*, *IL10*) markers. Each point in bar plot represents a single technical replicate from frozen normal human retroperitoneal adipose tissues (rpWAT, *n* = 3), well-differentiated liposarcoma (WD, *n* = 2), and dedifferentiated (DD, n = 2). Bar plot represents mean ± standard deviation of fold change when compared to rpWAT; *, *p* < 0.05; **, *p* < 0.001; ***, *p* < 0.0001. N/A, not available; UD, undetermined; AA/Black, African American/Black; W, White

RNA was obtained using RNAeasy Plus Mini (Qiagen; Cat#: 74134) according to manufacturer’s protocol. Isolated RNA was reversed transcribed using Applied Biosystems high-capacity cDNA reverse transcription. Real time PCR was performed with Taqman or SYBR green using Applied Biosystems 7900HT system. Results were normalized to one of the most stable gene found in liposarcoma, *IPO8*, [29, 30] and analyzed by the 2^-∆∆CT^ method calibrated with normal human retroperitoneal white adipose tissue (rpWAT) in Fig. 1 or pre-induction (Day 0) values of normal human retroperitoneal adipose primary cells (HuASC) in Figs. 4, 6 and 7. The primers are listed in Supplementary Materials Table S1.

### Immunoblot analysis

The normal adipose tissues or tumor tissues were homogenized in RIPA buffer and processed for immunoblotting. HuASC, WDLPS, and DDLPS cells were harvested at three different time points (0, 3, and 14 days) after induction for adipogenesis. The monolayer cells were rinsed with PBS and collected in RIPA buffer for immunoblotting. The extracts were sonicated on ice and complete nuclei lysis was confirmed by Trypan blue staining.

Proteins (25 μg) were separated in SDS-containing 10% polyacrylamide gels and transferred to nitrocellulose membranes. After transfer, membranes were blocked in 4% nonfat milk in 25 mM Tris-Cl (pH 8.0) with 150 mM NaCl and 0.1% Tween 20 for 1 h at room temperature. The membranes were incubated with anti-PPARG (Santa Cruz; Cat #: SC-7273; 1:250), anti-SIAH2 (LSBio; Cat #: LS-C112149; 1:1000), anti-ZFP423 (Millipore-Sigma; Cat#: ABN410; 1:500) or anti-ZFP521 (ProSci; Cat #: 6859; 1:1000) as indicated for 1–2 h at room temperature. The results were visualized with HRP-conjugated secondary antibodies (Jackson ImmunoResearch) and enhanced chemiluminescence (Pierce). Western blot images shown in Figs. 1, 5, 6 and 7 were obtained from SDS-PAGE trimmed to retain the entire resolving gel to ensure a molecular weight range from 25 to 250 kD or higher prior to hybridizing with the antibodies. When a panel of samples was duplicated on a single gel (Figs. 1, 6 and 7), the full-length nitrocellulose was trimmed on either side of each panel to allow detection of the panels with different antibodies. The images in the figures are cropped to show at least two molecular weight markers flanking the bands of interest. The corresponding images of replicate blots are shown in the supplementary Figs. S1-S4 that include the entire range of molecular weights from 25 to 250 kD. Note that the bands of interest for unmodified PPARG1 and PPARG2 are at 50kD with very high molecular weight bands also shown.

### In-situ hybridization, immunohistochemistry, and histological staining

Paraffin-embedded tissues were sectioned into 5 μm slices and hematoxylin and eosin (H&E) stained. *SIAH2* mRNA was detected by fluorescence in-situ hybridization (*SIAH2*) and co-localized with macrophages (anti-IBA1) or adipocytes (anti-PLIN1) using immunohistochemistry.

In-situ hybridization (ISH) was performed using RNAscope (ACD; Cat# 32100). Tissue sections were deparaffinized with xylene and dehydrated using ethanol. Protease treatment and heat-induced target retrieval followed the manufacturer’s recommendations. Sections were hybridized using a *SIAH2* probe (ACD; Cat. #490121) for 2 h at 40 °C. After hybridization, the sections were sequentially incubated with AMP1, AMP2, AMP3, and then C1-HRP to amplify the probe signal. The signal was detected using TSA Plus Fluorescence Kit (PerkinElmer; Cat#: NEL744E001KT).

Immunohistochemistry (IHC) immediately followed ISH. Anti-IBA1 or anti-PLIN1 antibodies were applied overnight at 4 °C. After the primary antibody, donkey-anti-rabbit Alexa 488 secondary antibody was applied for 1 h at room temperature. Sections were counterstained with DAPI in Prolong Gold Antifade Mountant (ThermoFisher; Cat# P36931) and analyzed by wide field Leica DMI6000 (Leica Microsystems) fluorescence microscopy at 20X and 40X objectives. The ratio of SIAH2 mRNA to nuclei was determined using the ImageJ Analyze Particle function in the corresponding imaging channels for SIAH2 mRNA signals or DAPI staining of nuclei.

### Statistical analysis

Statistical significance was determined using an unpaired two-tailed t-test with GraphPad Prism 8 software and reported as the mean ± standard deviation.

## Results

### Expression of adipogenic and myeloid markers in rpWAT, WDLPS and DDLPS tissues

We first analyzed gene and protein expression of adipogenic and myeloid cell markers in normal retroperitoneal white adipose tissue (rpWAT), WDLPS and DDLPS tumor tissues. Due to the rarity of these liposarcomas, only two fresh tissues were available for each type of liposarcoma (Fig. 1A). The adipocyte-specific PPARG2 protein is expressed in the rpWAT and WDLPS, but is absent in the DDLPS (Fig. 1B). Higher expression of the PPARG2 target genes and adipocyte markers *ADIPOQ* and *PLIN1* was also observed in the rpWAT and WDLPS compared to DDLPS (Fig. 1C). Along with upregulated markers of adipocyte formation in the WDLPS, adipocyte precursor cells are also present in the WDLPS, as indicated by *ZFP423, PDGFRA,* and *PREF1* in the WDLPS. The more broadly expressed *PPARG1* mRNA does not translate to PPARG1 protein expression or induction of adipogenic markers in the DDLPS (Fig. 1B, C).

Although *ZFP423* mRNA is elevated in the DDLPS relative to normal rpWAT, this does not translate into ZFP423 protein expression in the DDLPS (Fig. 1B). ZFP423 protein levels are substantially reduced in the DDLPS tissue compared to rpWAT and WDLPS tissues (Fig. 1B and Fig. S4), corresponding to the absence of the adipocyte-specific PPARG2 protein (Fig. 1B and Fig. S4) and significantly reduced *PPARG2* mRNA expression (Fig. 1C).

### SIAH2 mRNA expression is upregulated in DDLPS compared to rpWAT or WDLPS

In addition to increased ZFP423 mRNA in the DDLPS, *SIAH2*, *CEBPB*, and *CEBPD* mRNA are other markers of early adipogenesis that are elevated in DDLPS when compared to rpWAT and WDLPS in contrast to reduced *ZFP521* levels. (Fig. 1C). SIAH2 protein levels are elevated in the WDLPS and DDLPS, but this does not consistently occur in the limited tissue samples examined in the current study (Fig. 1B and Fig. S1).

Macrophages also express *SIAH2* (human tissue datasets in immgen.org) and play a key role in promoting adipose tissue expansion [31, 32] and tumorigenesis [33]. Therefore, we examined the gene expression of the markers for pan-macrophage (*CD11B, CD64*), a pro-inflammatory (*IL6*) and anti-inflammatory cytokine (*IL10*) (Fig. 1D). Even though *CD11B* mRNA expression was comparable between the tissues, *CD64* expression was increased in the DDLPS tissue. Unlike CD11B, CD64 distinguishes between adipose tissue macrophage and dendritic cells [34]. Increased *CD64* is consistent with higher macrophage levels in DDLPS tissues than normal rpWAT or WDLPS.

Liposarcoma tissues showed higher *IL6* expression with the highest *IL6* expression found in DDLPS tissue. WDLPS exhibited higher gene expression for the anti-inflammatory marker *IL10*. These data suggest macrophages recruitment and pro-inflammatory cytokine expression is elevated in DDLPS compared to normal human retroperitoneal adipose tissue or WDLPS.

**Fig. 2:**
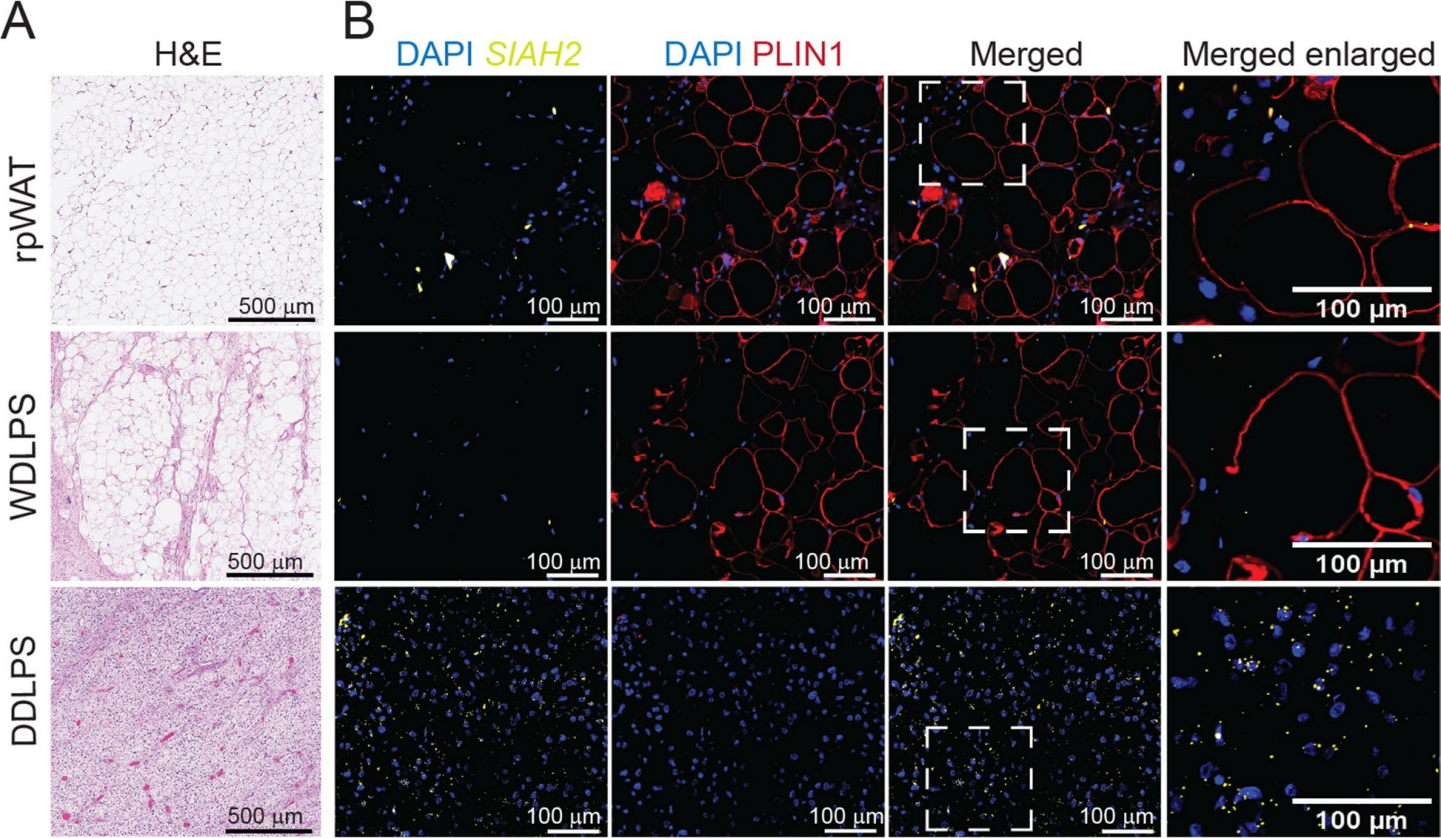
*SIAH2* mRNA is upregulated in DDLPS compared to WDLPS and rpWAT. **A** Hematoxylin and eosin (H&E) stained DDLPS, WDLPS, and rpWAT. **B** In situ hybridization detection of *SIAH2* (yellow) and immunohistochemistry detection of nuclei (DAPI, blue) and adipocytes using PLIN1 (red) in DDLPS, WDLPS, and rpWAT. Section of the image, marked by the white square, was enlarged to aide with *SIAH2* visualization. The images shown are representative of *N* = 3 for rpWAT, *N* = 4 for WDLPS and *N* = 5 for DDLPS

**Fig. 3:**
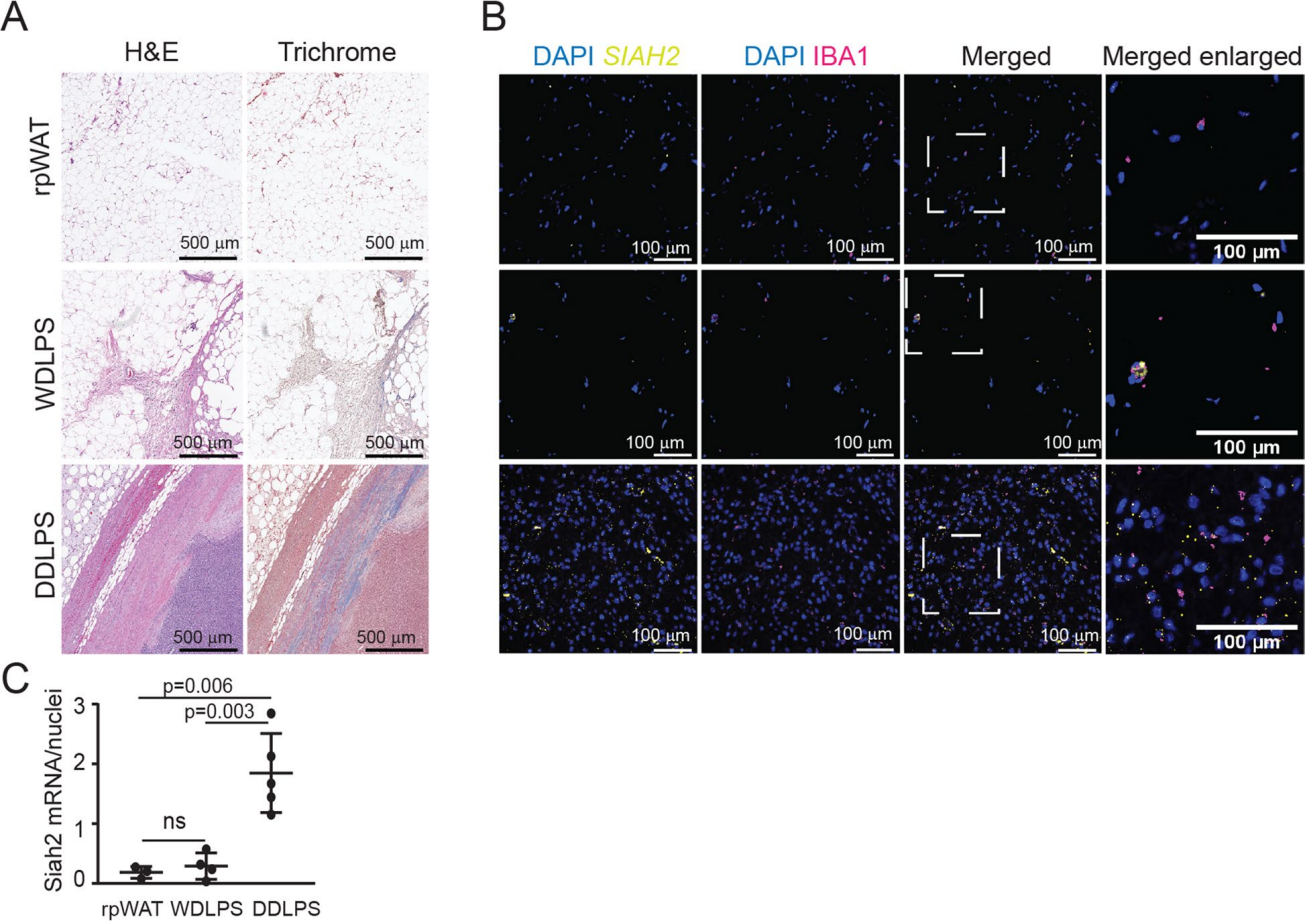
DDLPS is more fibrotic and heterogeneous with more macrophages compared to rpWAT or WDLPS tissues. **A** Hematoxylin and eosin (H&E) or trichrome stained DDLPS, WDLPS, and rpWAT. Trichrome stains connective tissue, an indication of fibrosis. **B** In situ hybridization detection of *SIAH2* (yellow) and immunohistochemistry detection of nuclei (DAPI, blue) and macrophages using IBA1 (magenta) in DDLPS, WDLPS, and rpWAT. Section of the image, marked by the white square, was enlarged to aide with *SIAH2* visualization. The images shown are representative of N = 3 for rpWAT, N = 4 for WDLPS and N = 5 for DDLPS. **C** The ratio of SIAH2 mRNA to nuclei in the images shown in Figs. 1 and 2B was determined using the ImageJ Analyze Particle function in the corresponding imaging channels for SIAH2 mRNA signals or DAPI staining of nuclei

As an independent approach to assay *SIAH2* mRNA expression in WDLPS or DDLPS tissues and possible co-localization with macrophages, we used paraffin-embedded tissues to examine *SIAH2* mRNA expression by in situ hybridization (ISH) in tandem with immunohistochemical (IHC) detection of lipid-laden adipocytes (perlipin-1, PLIN1, Fig. 2) or macrophages (ionized calcium binding adaptor molecule 1, IBA1, Fig. 3). Not surprisingly, liposarcoma tissues were more heterogeneous than normal retroperitoneal adipose tissues (rpWAT) as determined by H&E staining (Figs. 2 and 3A). Abundant perilipin-1 expression was observed from both rpWAT and WDLPS but not in DDLPS (Fig. 2B). This agrees with the reported adipogenic discrepancy between WDLPS and DDLPS [1, 4] and previous studies identifying perilipin-1 as a marker to differentiate between WDLPS and DDLPS [4, 35, 36]. Notably, when present, the adipocytes in the DDLPS tissues do not pack tightly as shown for the healthy rpWAT or the WDLPS (Fig. 3A). The increased heterogeneity in the morphology of the LPS tissues corresponds to increased fibrosis (trichrome stain, Fig. 3A) in WDLPS and DDLPS and higher levels of macrophages in DDLPS than in WDLPS or normal rpWAT tissues (Fig. 3B). *SIAH2* mRNA is expressed at substantially higher levels in DDLPS compared to normal rpWAT or WDLPS (Fig. 2B, Fig. 3B-C). This corresponds to *SIAH2* expression in the majority of the DDLPS stromal cells visualized. Moreover, *SIAH2* colocalizes with a subset of the stromal tumor-associated macrophages (IBA1) in the more fibrotic DDLPS (Fig. 3B). These observations agree with the gene expression patterns in the fresh tissues (Fig. 1B), where SIAH2 mRNA and macrophage markers are present at higher levels in the DDLPS.

### Adipogenic potential in liposarcoma cell lines corresponds to ZFP423 expression

**Fig. 4:**
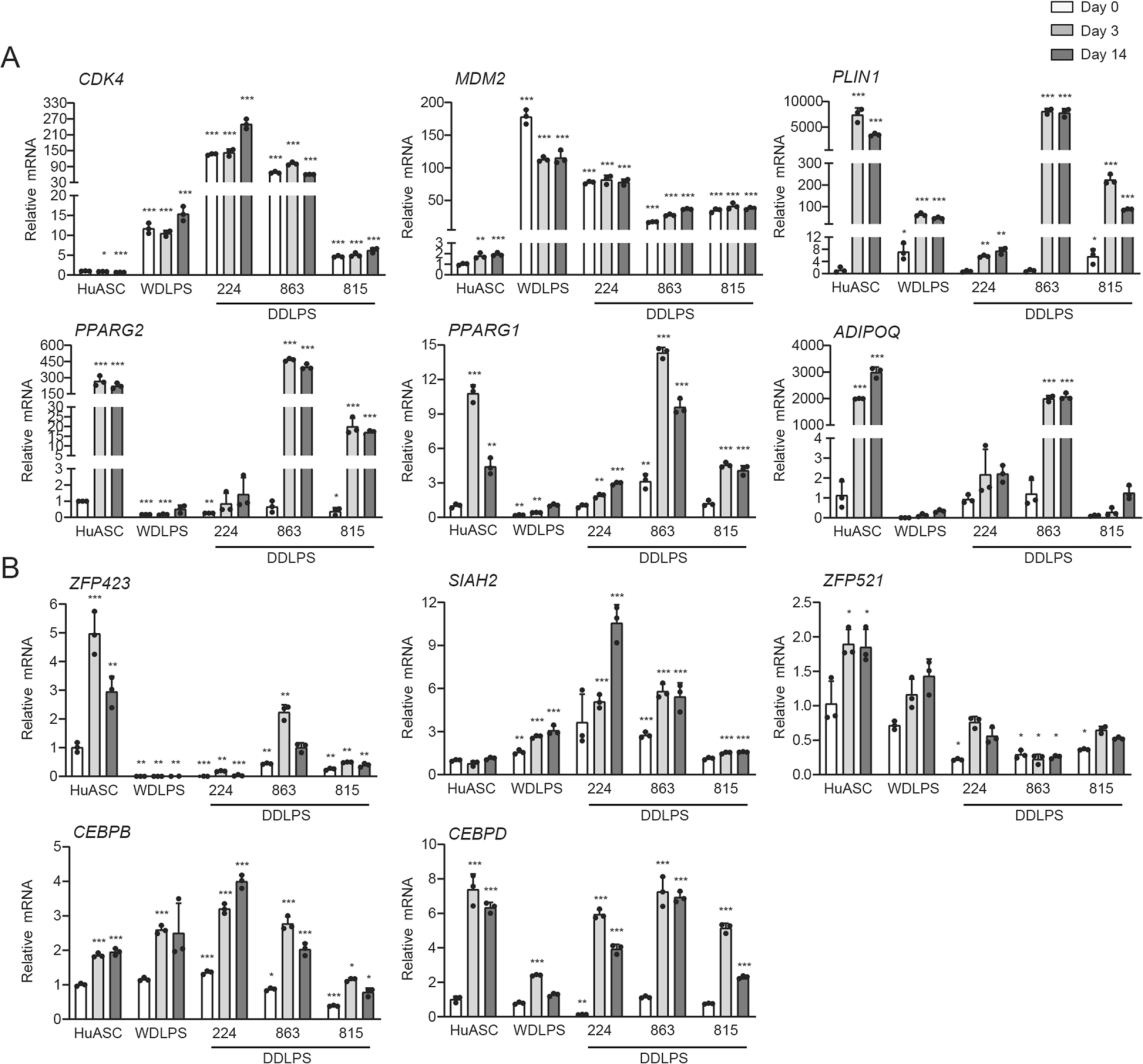
*ZFP423* mRNA levels correlate with *PPARG2* mRNA levels in normal white adipose tissue primary cells or WDLPS and DDLPS cell lines. Gene expression of biomarkers of WDLPS and DDLPS (*MDM2, CDK4*) and factors involved in adipogenesis of human retroperitoneal adipose primary cell (HuASC), WDLPS (WD), and DDLPS (224, 863, and 815) at pre-induction, day 3 of induction, and day 14 of induction for adipogenesis. Bar plots depict mean ± standard deviation compared to pre-induced HuASC; *, *p* < 0.05, **, *p* < 0.01, ***, *p* < 0.001. Each point in the plot represents a technical replicate. 224, Lipo224; 863, Lipo863; 815, Lipo815. The plots are representative of experiments that were repeated at least twice

To examine expression of *ZFP423*, *SIAH2* and *ZFP521* mRNA when WDLPS or DDLPS cells are induced to undergo adipogenesis, we assayed mRNA levels in primary stromal cells from normal retroperitoneal adipose tissue (HuASC), WDLPS cell line 94 T778 (ATCC) and DDLPS cell lines Lipo224, Lipo863, and Lipo815 [3, 37] exposed to an adipogenic cocktail. We first confirmed expression of C*DK4* and *MDM2* as established biomarkers of WDLPS and DDLPS [1] and determined that both markers were highly expressed in the liposarcoma cell lines regardless of adipogenic potential when compared to normal HuASC primary cells (Fig. 4A). Markers of adipogenesis (*PLIN1, PPARG1, PPARG2* and *ADIPOQ*) were upregulated in the HuACS and DDLPS Lipo863 (Fig. 4A), but substantially reduced in the WDLPS, DDLPS Lipo224, and DDLPS Lipo815 compared to HuASC and DDLPS Lipo863. Increased expression of *ZFP423* mRNA closely corresponded to induction of *PPARG2* mRNA (Fig. 4B). However, expression of genes encoding other factors regulating early steps in adipogenesis (*SIAH2, ZFP521, CEBPB,* and *CEBPD*) (Fig. 4B) did not correlate with *PPARG2* mRNA expression indicative of mature adipocyte formation in the cell lines. Thus, the adipogenic machinery is expressed in WDLPS, DDLPS Lipo224 and DDLPS Lipo815, but does not stimulate *PPARG2* mRNA expression in the absence of *ZFP423* mRNA.

**Fig. 5:**
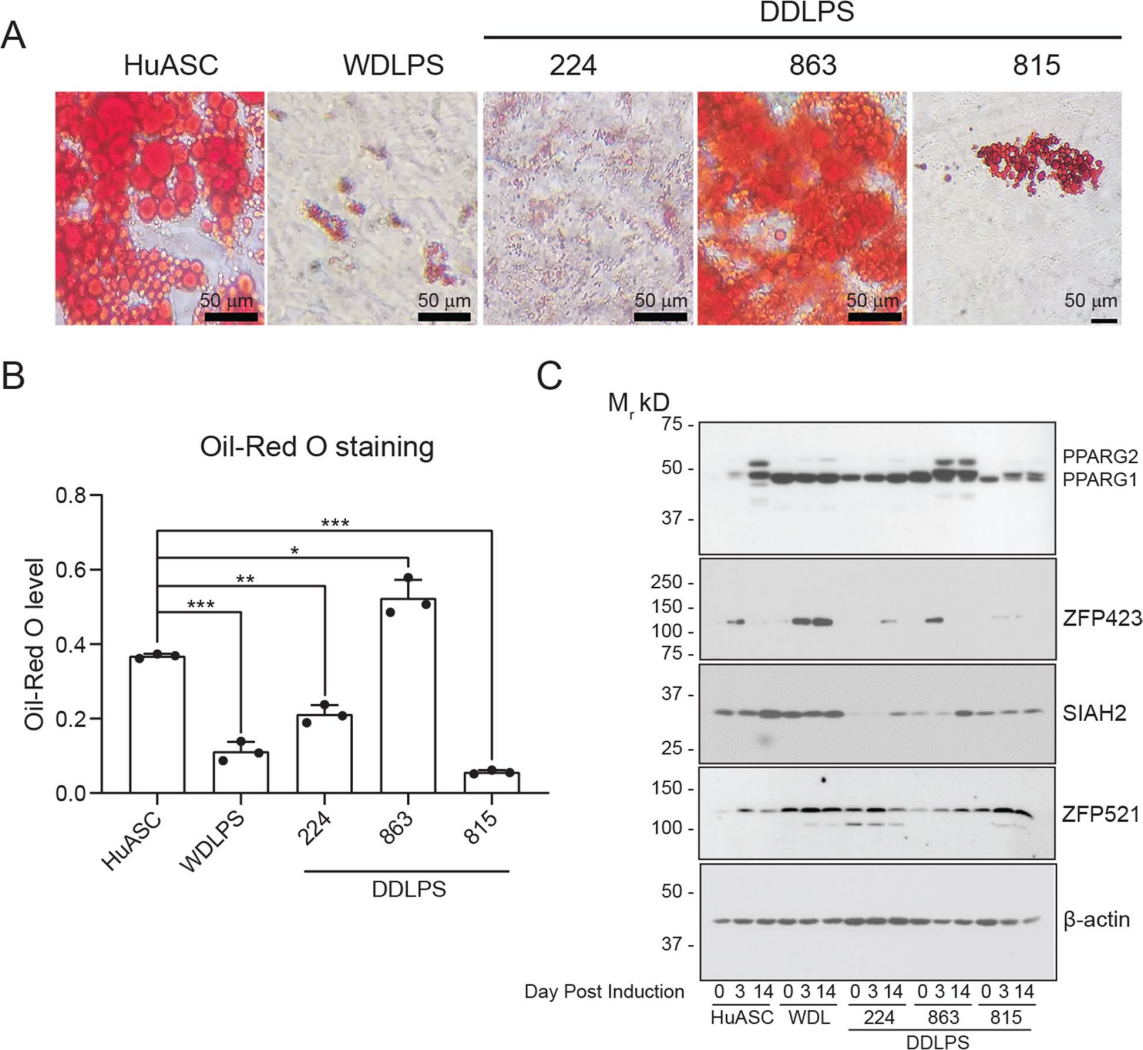
ZFP423 protein expression is associated with upregulation of PPARG2 protein in normal adipose tissue primary cells (HuASC) and DDLPS cell lines. **A** Oil Red O staining of human retroperitoneal adipose primary cell (HuASC), WDLPS (WD), and DDLPS (224, 863, and 815) after 14 days of adipogenic induction. **B** Quantification of Oil Red O staining. **C** Western blot analysis of ZFP521, SIAH2, PPARG, and β-actin from HuASC, WDLPS, and DDLPS cell lines at pre-induction (Day 0), and 3 (Day 3) or 14 days (Day 14) post induction. 224, Lipo224 cell line; 863, Lipo863 cell line; 815, Lipo815 cell line. A full-length, original and unprocessed version of the western blot for each antibody and densitometry ratio quantification is shown in Supplemental Fig. S2. The plots are representative of experiments that were repeated at least twice. Bar plots depict mean ± standard deviation compared to HuASC; *, *P* < 0.05, ** *P* < 0.1 and *** *P* < 0.001

To determine if *PPARG2* and *ZFP423* mRNA levels corresponded to forming mature adipocytes, we assayed neutral lipid accumulation in the cell lines using Oil Red O staining (Fig. 5A, B). The normal HuASCs and DDLPS Lipo863 cells readily formed mature adipocytes (Fig. 5A, B). In contrast, WDLPS, DDLPS Lipo224 and DDLPS Lipo815 cell lines were minimally induced to form the lipid droplets characteristic of adipocytes (Fig. 5A, B). Lipid accumulation corresponds to induction of the adipocyte-specific PPARG2 isoform in the HuASC and Lipo863, but only PPARG1 protein expression or low levels of PPARG2 in the remaining cell lines (Fig. 5C & Fig. S2). SIAH2 protein levels are generally lower in the HuASCs and DDLPS Lipo863 cells pre-induction but increase during induction when mature adipocytes are formed. ZFP521 levels are inversely related to SIAH2 levels at day 14 post induction in the HuASCs and are lower in the adipogenic DDLPS 863 compared to the less adipogenic WDLPS and DDLPS 224 and 815. ZFP423 levels increase at day 3 post-induction in the HuASCs and DDLPS Lipo863 preceding PPARG2 expression, but are minimally expressed in the non-adipogenic DDLPS Lipo224 or DDLPS Lipo815. Notably, ZFP423 protein is highly upregulated in the WDLPS cell line that failed to form mature adipocytes in the absence of ZFP423 mRNA induction and only minimal PPARG2 gene or protein expression, suggesting posttranslational changes in ZFP423 protein in the WDLPS cell line that affect ZFP423 transcriptional coactivator function.

### Regulation of *ZFP423* expression alters adipogenic potential in liposarcoma cell lines

**Fig. 6:**
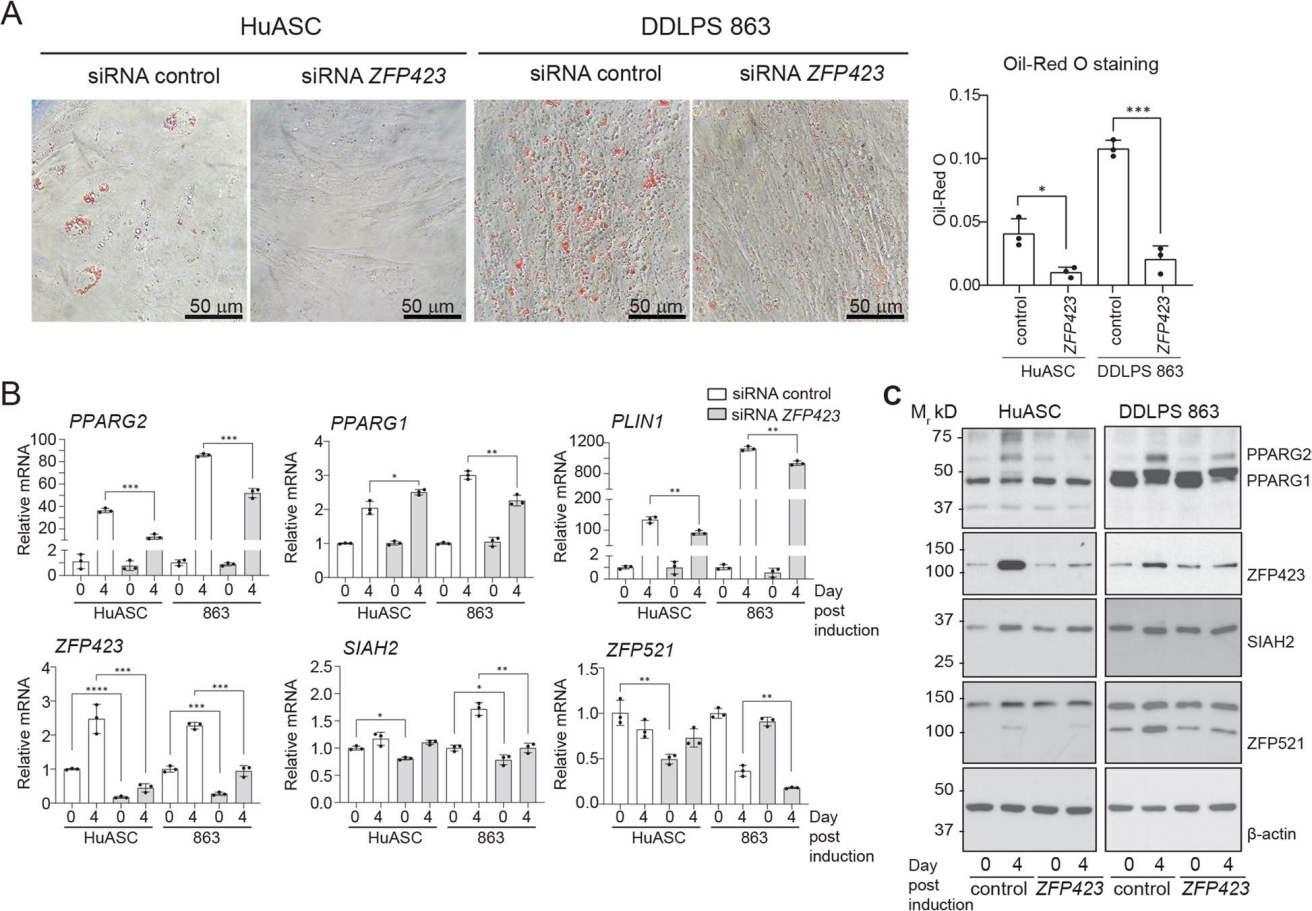
*ZFP423* knockdown attenuates adipocyte differentiation in human ASC and DDLPS Lipo863 cells. **A** Oil Red O staining of human retroperitoneal adipose primary cells (HuASC) and DDLPS (Lipo863) after 4 days of adipogenic induction with accompanying quantification of Oil Red O staining. **B** Gene expression of factors involved in adipogenesis at pre-induction (D0) and day 4 (D4) post-induction. **C** Western blot analysis of ZFP521, SIAH2, PPARG, ZFP423, and β-actin at pre-induction (0) and day 4 [4] post induction. Bar plots depict mean ± standard deviation compared to pre-induced HuASC; *, *p* < 0.05, **, *p* < 0.01, ***, *p* < 0.001. Each point in the plots (A and B) represents a technical replicate. Full-length western blot replicates and densitometry ratio quantifications are shown in Supplemental Fig. S3. Note that the western blots for PPARG1 and PPARG2 detection in S3 (native MW around 50 kD) include very high molecular weight bands that may represent ubiquitin-modified PPARG

The failure of the WDLPS cell line to form adipocytes is consistent with earlier reports that the immortalized WDLPS T778 cells derived from a recurring WDLPS are resistant to adipogenesis [37]. Our results with the DDLPS 863 cell line also agree with an earlier report that the cells express high levels of PPARG when grown in adipogenic conditions [3]. Although this response may reflect the culture conditions of the WDLPS T778 and DDLPS Lipo863 cell lines, we exploited the observed adipogenic potential in the WDLPS T778, DDLPS Lipo863 and DDLPS Lipo224 cell line to ask if modifying *ZFP423* expression is sufficient to alter adipogenesis in the liposarcoma cell lines. We first used a siRNA-based approach to deplete *ZFP423* expression in the HuASC and DDLPS Lipo863 cells that readily undergo adipogenesis. The time course was limited to 4 days post induction due to the transient nature of siRNA-mediated gene knockdown. Consistent with ZFP423’s critical role in promoting adipocyte differentiation, ZFP423 depletion significantly decreased lipid accumulation in both HuASC and DDLPS Lipo863 at 4 days post-induction (Fig. 6A). ZFP423 mRNA and protein expression was attenuated in both HuASC and DDLPS Lipo863 when exposed to the ZFP423 siRNA compared to the non-specific siRNA (Fig. 6B, C & Fig. S3). Although repressed compared to control, ZFP423 expression increased with adipogenic induction compared with pre-induction, suggesting possible loss of efficient *ZFP423* knockdown at 7 days post transient transfection. Reduced ZFP423 expression led to a significant decrease in *PPARG2* and *PLIN1* gene expression in the HuASCs and DDLPS Lipo863 cells at 4 days post-induction. *SIAH2*, *ZFP521* and *PPARG1*were reduced at day four post induction in the DDLPS Lipo863 cells with ZFP423 depletion, but SIAH2 and ZFP521 protein levels were unchanged.

**Fig. 7:**
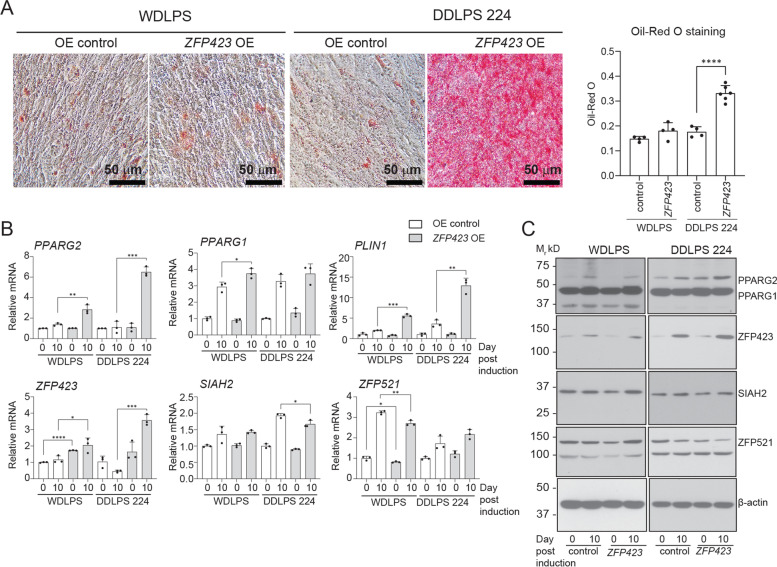
*ZFP423* overexpression (OE) promotes adipocyte differentiation in DDLPS Lipo224. **A** Oil Red O staining of well-differentiated liposarcoma (WDLPS) and DDLPS (Lipo224) after 10 days of adipogenic induction with accompanying quantification of Oil Red O staining. **B** Gene expression of factors involved in adipogenesis at pre-induction (D0) and day 10 (D10). **C** Western blot analysis of ZFP521, SIAH2, PPARG, ZFP423, and β-actin at pre-induction (0) and day 10 [10] post-induction. Bar plots depict mean ± standard deviation compared to pre-induced HuASC; *, *p* < 0.05, **, *p* < 0.01, ***, *p* < 0.001. Each point in the plots represents a technical replicate. 224, Lipo224. Full-length western blot replicates and densitometry ratio quantifications are shown in Supplemental Fig. S4. Note that the western blots for PPARG1 and PPARG2 detection in S4 (native MW around 50 kD) include very high molecular weight bands that may represent ubiquitin-modified PPARG

In parallel, overexpression of *ZFP423* was achieved using retroviral gene delivery into the WDLPS and DDLPS Lipo224 cell lines that have low *ZFP423* gene expression and poor adipogenic potential. *ZFP423* overexpression significantly promoted lipid accumulation in DDLPS Lipo224 but not WDLPS (Fig. 7A). At the gene level, *ZFP423, PPARG2* and *PLIN1* were upregulated in both WDLPS and DDLPS Lipo224 with *ZFP423* overexpression when compared to control (Fig. 7B). However, only DDLPS Lipo224 showed upregulated ZFP423 and PPARG2 protein expression (Fig. 7C). The pattern of PPARG1 gene and protein levels was unchanged by ZFP423 overexpression in the DDLPS Lipo224 (Fig. 7B, C and Fig. S4), in agreement with the specificity of ZFP423 toward PPARG2 in normal adipose tissue [14]. ZFP423 overexpression in the DDLPS Lipo224 cells did not affect SIAH2, and ZFP521 gene and protein expression as robustly as PPARG2, but protein levels of the anti-adipogenic ZFP521 are reduced with induction of adipogenesis when ZFP423 is overexpressed in the DDLPS Lipo224 cells. However, ZFP51 protein remains elevated in the relatively nonresponsive WDLPS cells. These results support a pivotal role for ZFP423 in determining the adipogenic potential of the dedifferentiated liposarcomas by regulating PPARG2 expression independent of changes in SIAH2 or ZFP521 expression.

## Discussion

The National Cancer Institute in the United States estimates that 13,460 new soft tissue sarcomas will be diagnosed and 5350 individuals will die of the disease in 2021 [38]. The majority of soft tissue sarcomas are adipose tissue-derived liposarcomas that have widely different subtype-dependent clinical outcomes. The most common subtype, well-differentiated liposarcoma (WDLPS) can dedifferentiate from the characteristic adipocyte-laden phenotype to the low-adipocytic dedifferentiated liposarcoma (DDLPS), an aggressive subtype associated with a significantly lower 5-year survival rate compared to WDLPS [39]. Both subtypes are characterized by highly amplified expression of MDM2 and CDK4, making inhibition of these proteins an attractive therapeutic target in the treatment of DDLPS and WDLPS [40, 41].

However, there are limitations to targeting MDM2 and CDK4. Apart from functioning to regulate cell cycle progression [42], CDK4 promotes adipocyte formation via directly interacting with PPARG to stimulate PPARG activity [43]. Although MDM2 is best known as regulating the activity of the tumor suppressor p53, MDM2 also promotes adipogenesis by activating a subset of cAMP-stimulated genes, particularly CEBPD [44]. These studies indicate that the disparity in mortality rates between WDLPS and DDLPS is driven by tumor biology independent of *MDM2* or *CDK4* amplification, pointing out that our current understanding of WDLPS and DDLPS does not fully address the molecular mechanisms underlying the developmental differences between the two liposarcomas that can be leveraged as therapeutic targets.

An alternate method of LPS classification relies on the tumors’ developmental/differentiation status. In this scheme, WDLPS is the most developmentally mature subtype, thus accounting for its higher adipose tissue composition. In a study of adult stem cells derived from the four LPS subtypes, Matushansky et al [45] showed that stromal cells taken from DDLPS retain stem cell-like markers, but the gene expression pattern of WDLPS stromal cells more closely resembles normal adipogenesis. Moreover, the Tontonoz et al study [5] showed *PPARG* mRNA expression was highly variable in human liposarcoma tissues. Reduced *PPARG* mRNA levels in high grade liposarcoma tissues supports the possibility that, in DDLPS, terminal differentiation is blocked at the early steps of mesenchymal cell commitment to adipogenesis that initiate PPARG2 expression rather than post-PPARG2 induction. These findings predict the early molecular events controlling the proliferation and conversion of adipocyte precursor cells to mature adipocytes may be differentially regulated between WDLPS and DDLPS.

In the current study, the strong positive relationship between *ZFP423* and *PPARG2* mRNA expression and induction of adipogenesis emerges in the cell culture experiments persists in the pattern of ZFP423 and PPARG2 protein expression in tissues. Although *CEBPB* and *CEBPD* are expressed early in adipogenesis and promote *PPARG2* mRNA expression [46, 47], induction of either gene is insufficient to drive *PPARG2* mRNA upregulation in the liposarcoma tissues in the absence of ZFP423 protein. Given ZFP423’s role as a transcriptional coactivator of *PPARG2* mRNA expression in determining preadipocyte commitment to adipogenesis in normal adipose tissue [14] and the impact of ZFP423 depletion or overexpression on adipogenic potential in the liposarcoma cells lines, these results suggest the difference in adipogenic capacity of the well-differentiated or dedifferentiated liposarcomas occurs at preadipocyte commitment.

A defect in preadipocyte commitment is also suggested by the absence of ZFP423 or PPARG2 protein in DDLPS tissues despite *ZFP423* mRNA expression in DDLPS comparable to WDLPS tissue. This corresponds to substantially reduced levels of preadipocyte factor-1 (*PREF1*) mRNA in DDLPS tissue compared to WDLPS. PREF1 is expressed earlier than ZFP423 and PPARG in highly proliferative adipocyte precursor cells that arise from mesenchymal progenitor cells [48]. But ZFP423 is also found in committed preadipocytes that are endothelial or pericyte in origin. This raises the possibility that ZFP423 and PPARG expression in a subset of adipocyte precursor cells of mesenchymal origin marked by *PREF-1* represent a critical pool of adipocyte precursor cells that is deficient in DDLPS.

Although the current study demonstrates a role for ZFP423 in determining the adipogenic potential of DDLPS tissues, the data do not delineate a role for ZFP521 or SIAH2 Cell lines with high adipogenic potential (HuASC and Lipo863) exhibited low ZFP521 protein expression, indicating ZFP521, an anti-adipogenic regulator of *ZFP423* expression [17] may act as a focal point for distinguishing between WDLPS and DDLPS adipogenic difference by regulating ZFP423 expression.

Tumor heterogeneity in the tissues may account for the variability in SIAH2 protein levels, but we also noted that *SIAH2* mRNA is consistently expressed at higher levels in the DDLPS tissues compared to normal retroperitoneal adipose tissue or WDLPS tissues. This raises the possibility of posttranscriptional modifications that alter the relationship between SIAH2 mRNA and protein levels in the liposarcomas [49]. Alternatively, SIAH2 is expressed in a substantial number of stromal cells present in the DDLPS, including tumor-associated macrophage that are abundant in DDLPS [50]. Notably, the macrophage-produced pro-inflammatory cytokine interleukin-6 (IL6) is significantly upregulated in the DDLPS tissue. In patients with soft tissue sarcomas, elevated circulating levels of IL6 predict the poor prognosis [51] typical of DDLPS. IL6 signaling in tumor-associated macrophage enhances tumor cell survival in hypoxia conditions more prevalent in DDLPS than WDLPS [52] that promote tumor progression and resistance to therapy [53, 54]. As a key regulator of hypoxia responses in normal and cancer cells [23], SIAH2 is positively correlated with metastasis progression [20, 23] that promotes tumor-associated macrophage recruitment in the hypoxic tumor microenvironment [55]. Thus, expression of *SIAH2* in DDLPS tumor-associated macrophage and other stromal cells indicates *SIAH2* expression may serve as a molecular marker distinguishing between DDLPS and WDLPS as well as a therapeutic target in DDLPS similar to melanoma, prostate, and breast cancer [56–58].

A more complete evaluation of the role of SIAH2 or ZFP521 in the DDLPS phenotype is limited by the availability of fresh tissues from these rare cancers. The cells lines obtained from liposarcomas present another limitation. While *MDM2* and *CDK4* mRNA as markers of WDLPS and DDLPS are reproduced, the cell lines do not reliably reflect in vivo behavior. This was previously reported by Peng et al. and Stratford et al. [3, 37], who isolated and initially characterized these cell lines. The low adipogenic potential of the WDLPS (94 T778) cell line and high propensity to undergo adipogenesis in the DDLPS (Lipo863) cell line suggest that fully understanding the developmental differences between WDLPS and DDLPS will require experimental conditions that account for the factors present in the tumor microenvironment. Despite these limitations, a clear relationship emerges between ZFP423 and PPARG2 protein expression in the phenotypic differences in well-differentiated and dedifferentiated liposarcomas.

## Conclusions

Our study provides novel insights into factors that determine the adipogenic potential of WDLPS and DDLPS. To our knowledge, our study is the first to identify dysregulation of pro-adipogenic factors regulating adipocyte precursor commitment to adipogenesis in dedifferentiated liposarcoma. These results point to additional targets related to adipogenesis in DDLPS to pursue in conjunction with ligand-activation of PPARG in committed preadipocytes, particularly regulation of ZFP423 in the DDLPS tissue. The current results also point to a possible role for the oncogenic ubiquitin ligase *SIAH2* mRNA as a biomarker of DDLPS.

## Supplementary Information

**Additional file 1: Supplementary Material Table S1** Provides the gene ID, accession number, primer sequences and primer manufacturer for all gene expression assayed in the current study. The information is categorized according to SYPR Green or Taqman chemistry. **Supplementary Material Figure S1-S4** Provide full blots and densitometry quantifications of western analysis found in Figs. 1, 5, 6, and 7.

## Data Availability

All data generated or analysed during this study are included in this published article and its supplementary information files.

## Abbreviations

ADIPOQ: Adiponectin
CEBPA: CCAAT-enhancer-binding protein alpha
CEBPD: CCAAT-enhancer binding protein delta
CDK4: Cyclin dependent kinase 4
DDLPS: Dedifferentiated liposarcoma
HuASC: Human adipose stromal cells
IL6: Interleukin 6
IL10: Interleukin 10
MDM2: Mouse double minute homolog 2
PDGFRA: Platelet-derived growth factor receptor alpha
PREF1: Preadipocyte secreted factor 1
rpWAT: Retroperitoneal white adipose tissue
SIAH2: Seven-in-absentia mammalian homolog 2
WDLPS: Well differentiated liposarcoma
ZFP432: Zinc finger protein 423
ZFP521: Zinc finger protein 521
