# Supplementary material for: Expression of the preadipocyte marker ZFP423 is dysregulated between well-differentiated and dedifferentiated liposarcoma
Source: BMC Cancer. 2022 Mar 21;22:300. doi: 10.1186/s12885-022-09379-6 (PMC8939188; doi:10.1186/s12885-022-09379-6)
Supplement: Supplementary file 1 — Additional file 1: Supplementary Material Table S1 Provides the gene ID, accession number, primer sequences and primer manufacturer for all gene expression assayed in the current study. The information is categorized according to SYPR Green or Taqman chemistry. Supplementary Material Figure S1-S4 Provide full blots and densitometry quantifications of western analysis found in Figs. 1, 5, 6, and 7. [file 12885_2022_9379_MOESM1_ESM.pdf]

A. SYBR Green

Gene ID	Gene Name	Accession #	Manufacturer	Sequence - Forward	Sequence - Reverse
CDK4	Cyclin Dependent Kinase 4	NM_000075.4	IDT	ACCCTGGTGTTTGAGCATGTAGAC	AAACTGGCGCATCAGATCCTTGA
MDM2	E3 Ubiquitin-Protein Ligase Mdm2	NM_002392.5	IDT	AGGAGATTTGTTTGGCGTGC	TGAGTCCGATGATTCCTGCTG
PLIN1	Perilipin 1	NM_002666.5	IDT	TTCAGTGAGGTAGCAGCCCT	CTGCAGGTGCCCATGTCACA
PPARG1	Peroxisome proliferator activated receptor gamma 1	NM_138712.3	IDT	GGCCGCAGATTTGAAAGAAG	CTGTGTCAACCATGGTCATTTC
PPARG2	Peroxisome proliferator activated receptor gamma 2	NM_015869.4	IDT	TCTCCTATTGACCCAGAAAGC	TCAACCATGGTCATTTCTTGTG
ADIPOQ	Adiponectin	NM_001177800.1	IDT	GATCCAGGTCTTATTGGTCCTAAG	CTCCTTTCCTGCCTTGGATT
SIAH2	Seven in Absentia Homolog 2	NM_005067.7	IDT	CAGTCCTGTTTCCCTGTAAGTATG	CAGGAGTAGGGACGGTATTCA
EBF1	Early B-cell factor 1	NM_001324109	IDT	ACAGCAATGGGATAAGGACGG	TGAGCAAGACTCGGCACATT
ZFP521	Zinc finger protein 521	NM_001308225	IDT	GTCGGATGAGAAGAAGACCT	GTTCAGTCCTTCATCAATCA
ZFP423	Zinc finger protein 423	NM_015069	IDT	AGTGCCCCGGAAAAAGACAT	ACTCGTGGTTGATGCCTTCC
PDGFRA	Platelet Derived Growth Factor Receptor Alpha	NM_006206.6	IDT	GGAAACAGAAACCGAGGTATGA	CTGCATCGGGTCCACATAAA
PREF1	Delta Like Non-Canonical Notch Ligand 1	NM_003836.6	IDT	GCTCTGTGATAGAGATGTTCGG	GGCACAGGAGCATTCATAGA
CD11B	Integrin subunit alpha M	NM_001145808.1	IDT	CAGAACAACCCTAACCCAAGAT	AACAGCTCTCGTACCACTTTG
CD64	Fc fragment of IgG receptor Ia	NM_000566.3	IDT	CTCCTTTGGGTTCCAGTTGAT	AAGGTTACGGTTTCCTCTTGG
IL6	Interleukin 6	NM_000600.5	IDT	AAAGAGGCACTGGCAGAAA	CAGGCAAGTCTCCTCATTGAA
CD206	Mannose Receptor C-type 1	NM_002438	IDT	GCTGATGGACTTCCTGGTAAC	CAGGACTCTGGATTGGACTTAAC
IL10	Interleukin 10	NM_000572.3	IDT	GATGCCTTCAGCAGAGTGAA	AACCCAGGTAACCCTTAAAGTC
IPO8	Importin 8	NM_006390.3	IDT	CATGATGCCTCTCCTGCATAA	GCATCTCCACATAGTACCTTCC

B. Taqman

Gene ID	Gene Name	Accession #	Manufacturer	Sequence - Probe	Sequence - Forward	Sequence - Reverse
CEBPB	CCAAT enhancer binding protein beta	NM_005194	IDT	AACTCTCTGCTTCTCCCTCTGCC	AGAAACGTCTATGTGTACAGATGA	GATTGCATCAACTTCGAAACCG
CEBPD	CCAAT enhancer binding protein delta	NM_005195	IDT	CGCTCCTATGTCCCAAGAAACTGCA	CAACGACCCATACCTCAGAC	GTAAGTCCAGGCTGTAGCTTC

Table S1: List of primers. A) SYBR primer sets. B) Taqman probes.

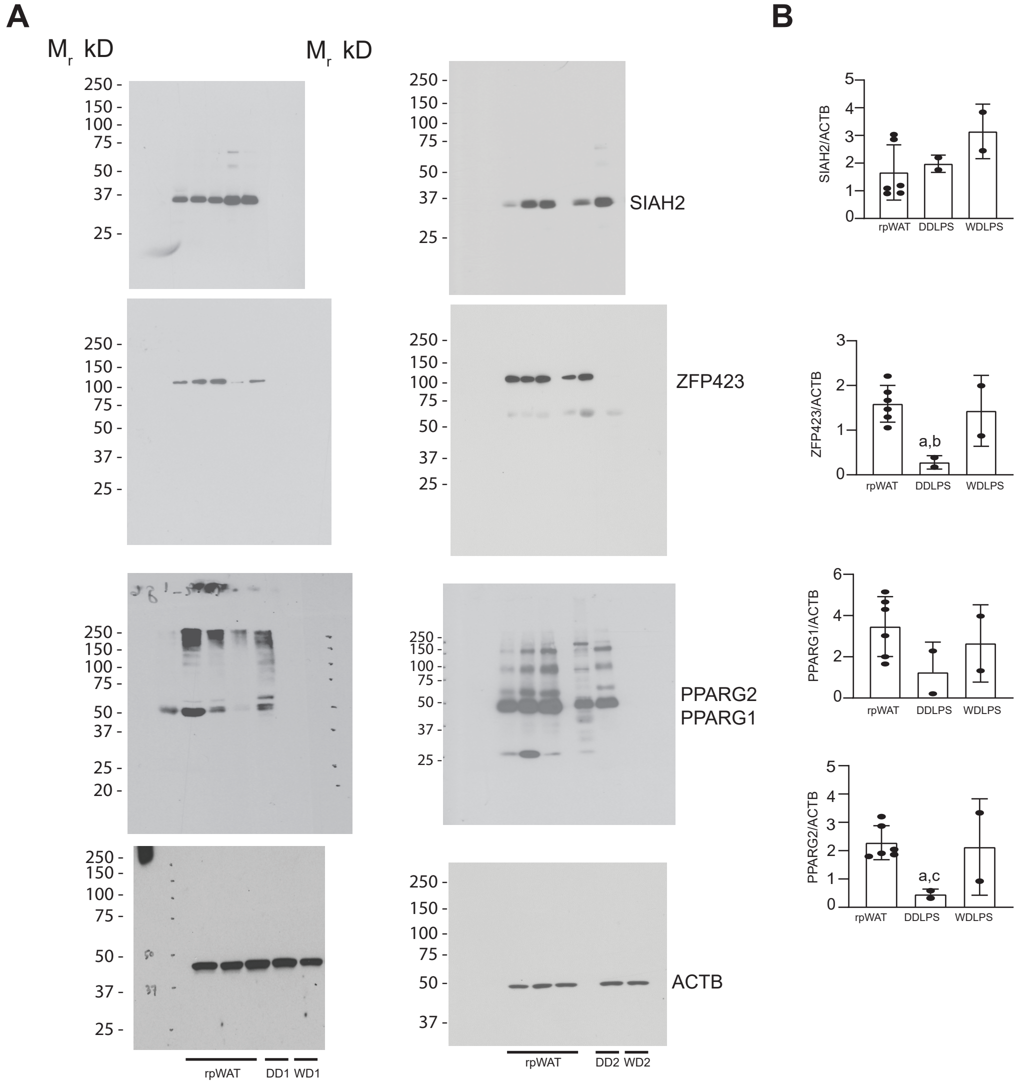

Figure S1: Uncropped western blots from Figure 1 and biological replicates of rpWAT, DDLPS, WDLPS. A) Full western blots. B) Densitometry ratio quantification of western blots when the protein of interest is compared to beta-actin (ACTB). Statistical difference shown as mean -/+ Std; (a)=p<0.01, DDLPS compared to rpWAT; b=p<0.05, DDLPS compared to WDLPS; c, p=0.11 for DDLPS compared to WDLPS.

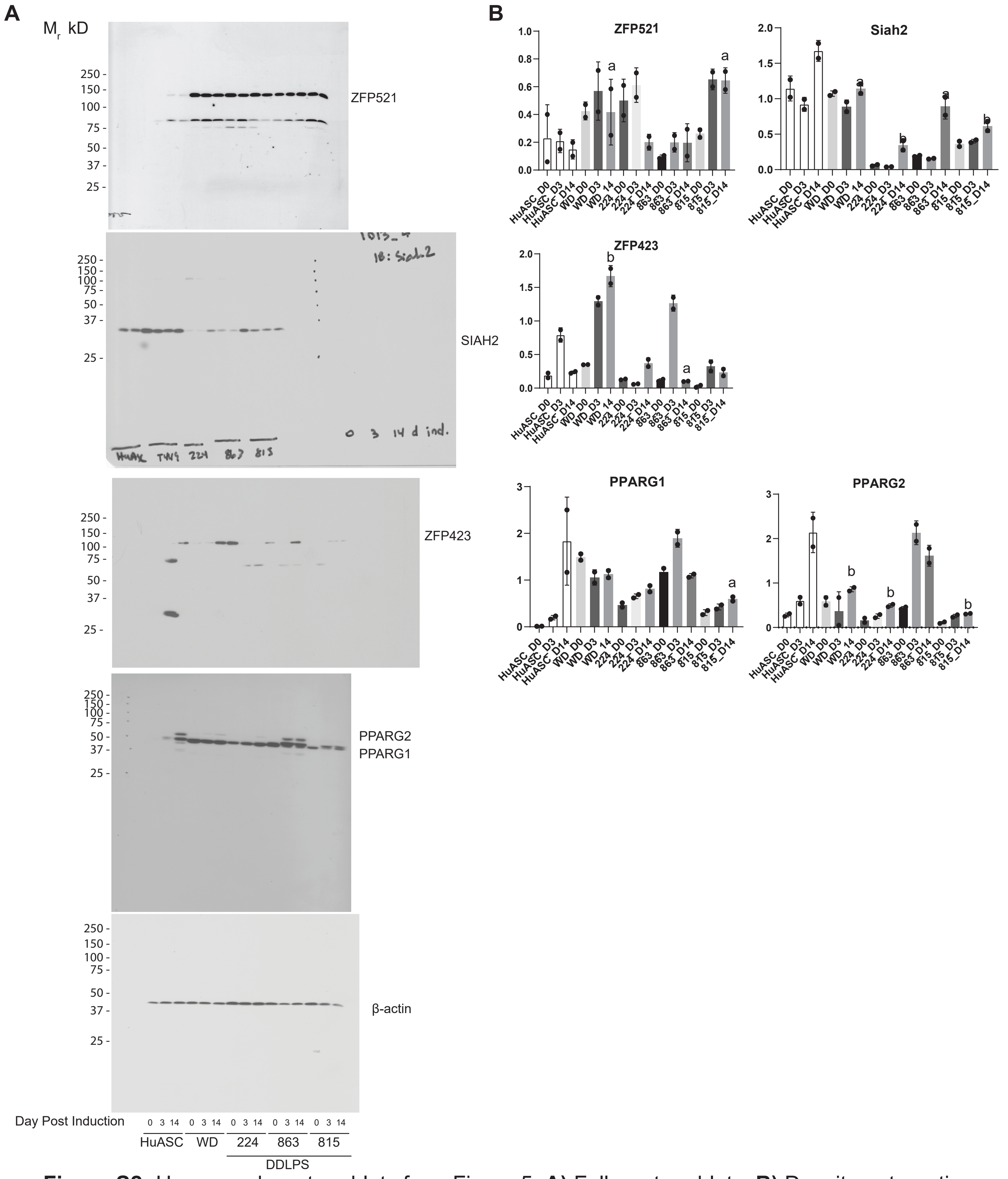

Figure S2: Uncropped western blots from Figure 5. **A)** Full western blots. **B)** Densitometry ratio quantification of replicate western blots. Statisitical significance is reported as the mean -/+ Std. a, p<0.05 compared to HuASC, day 14; b, P<0.01 compared to HuASC, day 14.

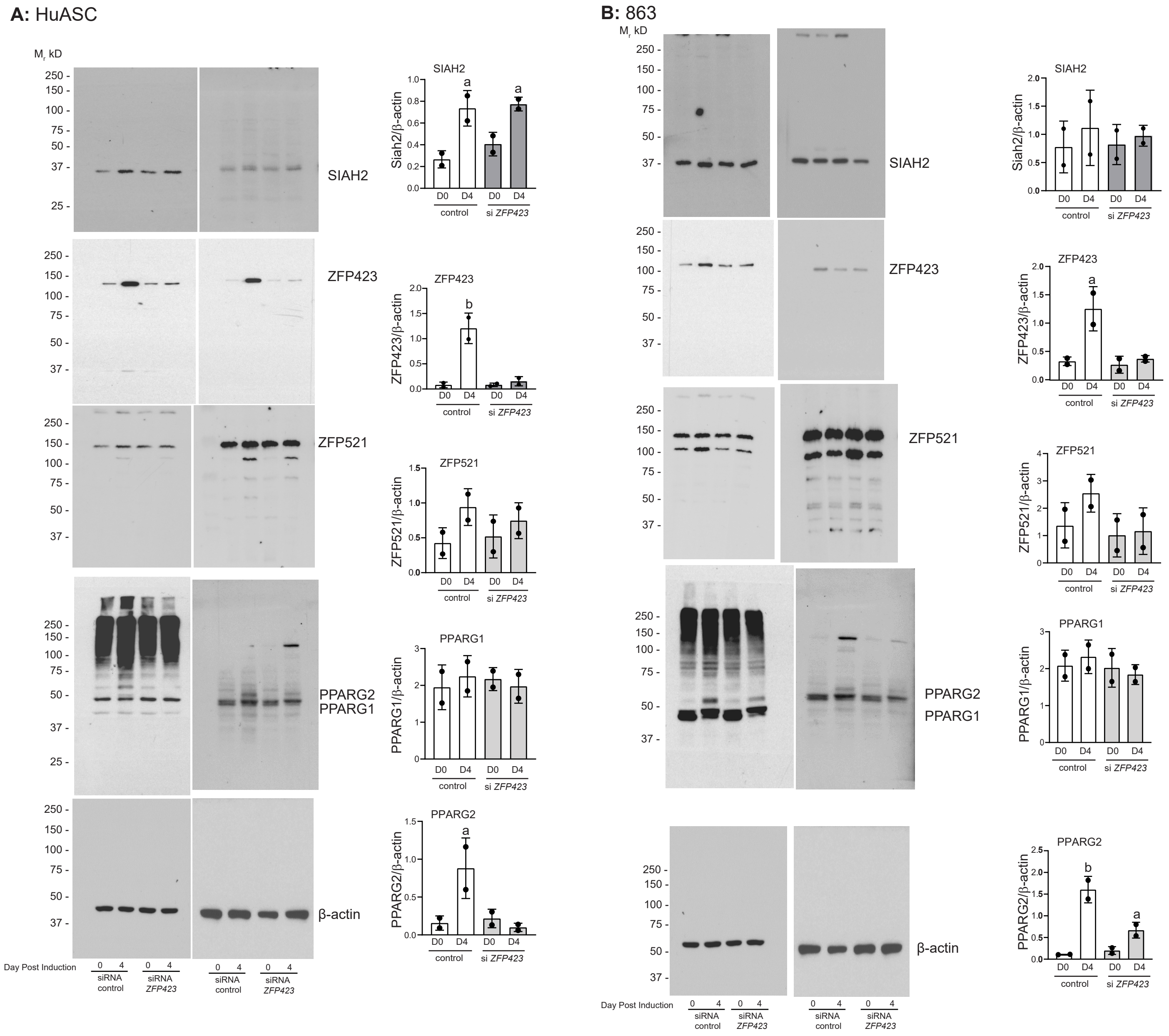

Figure S3: Uncropped western blots from Figure 6. A) Full western blot and densitometry of HuASCs. B) Full western blot and densitometry of DDLPS 863. Statistical differences are shown as the mean -/+ Std. a, p<0.05 compared to the related DO; b, p<0.01 compared to the related D0.

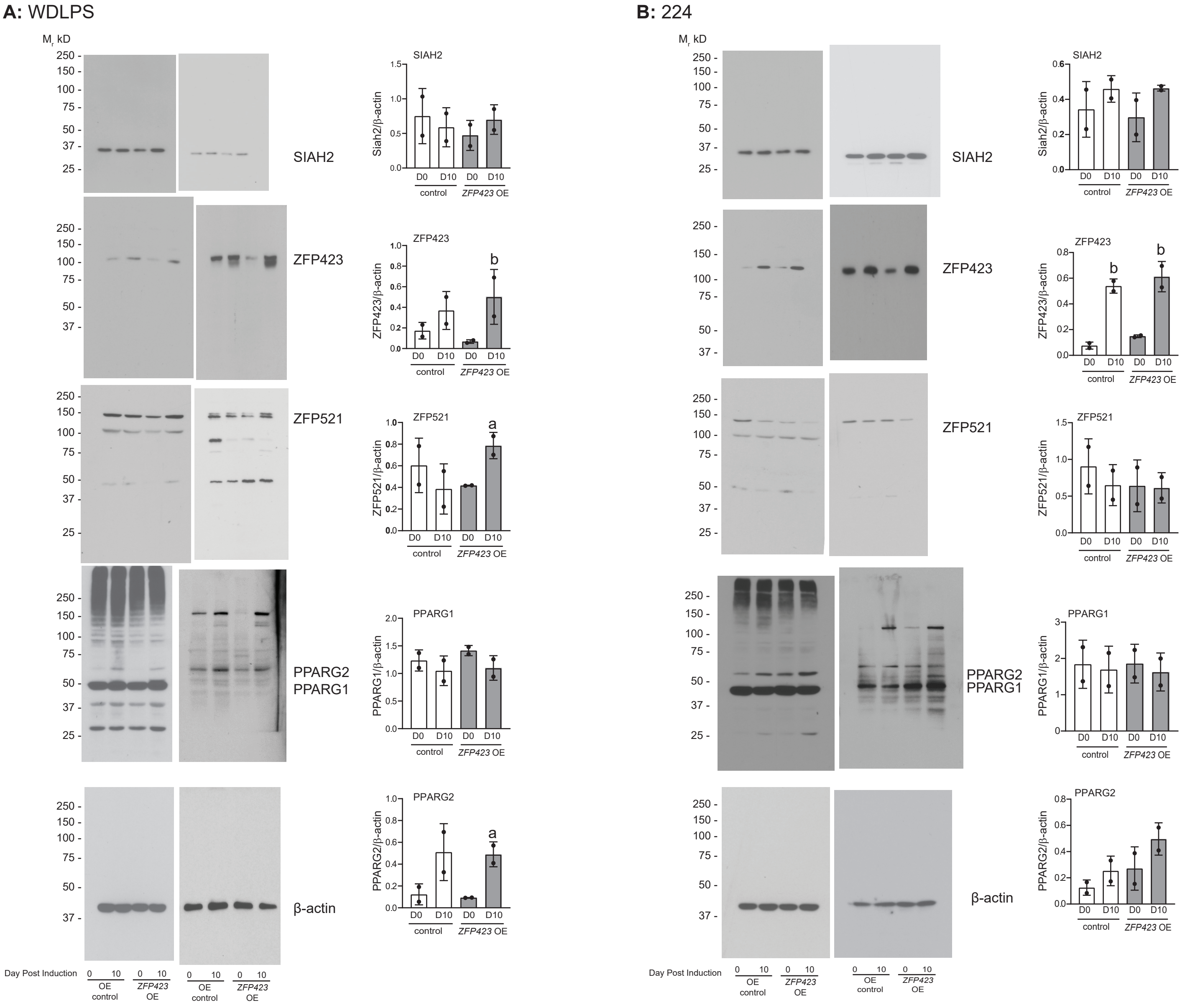

Figure S4: Uncropped western blots from Figure 7. A) Full western blot and densitometry of WDLPS. B) Full western blot and densitometry of DDLPS 224. Statistical differences are shown as the mean -/+ Std. a, p<0.05 compared to the related D0; b, p<0.01 compared to the related D0.
